# Ambient temperature and non-accidental mortality: A nationwide space–time-stratified case-crossover study within the 100 million Brazilian Cohort

**DOI:** 10.1016/j.envint.2025.109892

**Published:** 2025-11-04

**Authors:** Taísa R. Cortes, Julia M. Pescarini, Otavio T. Ranzani, Enny S. Paixão, Danielson J.D. Neves, Poliana Rebouças, Rita de Cássia Ribeiro-Silva, Andrêa J.F. Ferreira, Luiz A.C. Galvão, Elizabeth B. Brickley, Liam Smeeth, Ludmilla V. Jacobson, Ismael H. Silveira, Mauricio L. Barreto

**Affiliations:** a Center of Data and Knowledge Integration for Health (CIDACS), Oswaldo Cruz Foundation, Rua Mundo, 121, 41745-715 Salvador, Brazil; b Faculty of Epidemiology and Population Health, London School of Hygiene and Tropical Medicine, Keppel St, WC1E 7HT London, United Kingdom; c DataHealth Lab, Institut de Recerca Sant Pau (IR SANT PAU), Carrer de Sant Quintí, 77-79, 08041 Barcelona, Spain; d ISGlobal, Carrer del Doctor Aiguader, 88, 08003 Barcelona, Spain; e School of Nutrition, Federal University of Bahia (UFBA), R. Basílio da Gama, sn, 40110-907 Salvador, Brazil; f International Relations and Global Health Center, Fiocruz, Av. Brasil, 4365, 21040-900 Rio de Janeiro, Brazil; g Institute of Mathematics and Statistics, Fluminense Federal University, Rua Professor Marcos Waldemar de Freitas, s/n, 24210-201 Niteroi, Brazil; h Institute of Collective Health, Federal University of Bahia (ISC/UFBA), R. Basílio da Gama, sn, 40110-907 Salvador, Brazil

**Keywords:** Ambient temperature, Mortality, Vulnerability, Case-crossover study, Brazil

## Abstract

Research on ambient temperature effects on mortality leveraging cohort data remains scarce. This study estimates the association between ambient temperature and non-accidental mortality and investigates potential effect modification of the temperature-mortality relationship by a wide range of individual and household-level characteristics in a nationwide cohort. We designed a space–time-stratified case-crossover study within the 100 Million Brazilian Cohort, comprising low-income families enrolled in Brazil’s Unified Registry for Social Programs (CadÚnico), including all deaths from 2000 to 2018. Daily mean temperatures were obtained from Brazilian Daily Weather Gridded Data based on 1,252 meteorological stations. The outcome was defined as daily counts of non-accidental deaths aggregated at the municipality level. The effect of ambient temperature on non-accidental mortality was estimated using conditional quasi-Poisson regression combined with distributed lag non-linear models. The study population included 3,554,422 deaths. Cold temperatures were associated with a 38 % increase in non-accidental mortality risk (RR = 1.38, 95 % CI: 1.34–1.43) nationally, with high regional variability. In contrast, heat was associated with a 4 % increase in mortality risk nationally, primarily driven by increased risk in the Southeast and Central-West regions. Across population subgroups, cold-related mortality risk was higher among older (≥60 years) adults (RR = 1.47, 95 % CI: 1.42–1.53) and Black individuals (RR = 1.56, 95 % CI: 1.49–1.64). Higher vulnerability to heat-related mortality was found among urban residents (RR = 1.07, 95 % CI: 1.05–1.10). However, we found no consistent evidence of effect modification of cold- and heat-related mortality by sex, income or access to key utilities (water, waste, and electricity). Our findings indicate significant regional differences, highlighting population strata particularly vulnerable to temperature-related mortality. This evidence informs the targeting of interventions for protecting vulnerable populations from extreme temperatures.

## 1 Introduction

Numerous studies have investigated the effects of ambient temperature on mortality and morbidity, revealing a consistent pattern of increased risk during and after extreme temperature events. (Abbass et al., 2022; Green et al., 2019; Son et al., 2019) The literature also indicates that certain population groups are more vulnerable to temperature-related illnesses, including the elderly, children, and low socioeconomic groups. (Son et al., 2019; Benmarhnia et al., 2015) This increased risk is often attributed to disparities in exposure, socioeconomic conditions, and access to health services.

While research on temperature-related mortality has grown sub-stantially in recent decades, most of the evidence originates from populations in North America, Europe, and parts of Asia. (Green et al., 2019; Son et al., 2019) Given that the determinants of temperature-related health disparities vary across different settings and climatic conditions, investigations in tropical and low- to middle-income countries are essential for designing context-specific adaptation strategies.

In Brazil, a growing body of research has investigated the effects of temperature on mortality. (Aschidamini and Ponce de Leon, 2025; Bakhtsiyarava et al., 2023; Kephart et al., 2022; Jacobson et al., 2021; Silveira et al., 2019) Nevertheless, research investigating temperature-mortality heterogeneity in Brazil has largely depended on contextual indicators at city or regional levels, with few investigations assessing individual-level factors such as income and residential characteristics, partly due to their limited availability in national health databases. Additionally, analyses of temperature-related mortality focusing on socially and economically disadvantaged populations are limited.

Investigations on the effects of ambient temperature on historically marginalized populations, such as Black people and those living in extreme poverty, are crucial for the formulation of public health policies and designing climate change adaptation strategies that are effective and equitable. This takes on special relevance in a country such as Brazil, with entrenched socioeconomic inequalities and heterogeneous climate and built environments.

In the present study, we designed a space–time-stratified case-crossover study within the 100 Million Brazilian Cohort (100 M Cohort) to investigate vulnerability to the effects of temperature on non-accidental mortality. The 100 M Cohort provides extensive information on socially and economically disadvantaged populations in Brazil, (Barreto et al., 2022) offering a unique opportunity to investigate differences in the temperature-mortality relationship by individual and household characteristics.

## 2 Methods

### 2.1 Study design and population

We conducted a space–time-stratified case-crossover study including all individuals from the 100 M Cohort, (Barreto et al., 2022) across all age groups, who died from non-accidental causes between 2000 and 2018. The 100 M Cohort includes linked health and socioeconomic data on individuals belonging to low-income families enrolled in the CadÚ-nico – an electronic information system provided by the Brazilian federal government for registering families and individuals for receiving social benefits, including cash transfers. (Brasil, 2017).

The study population includes members of families with a per capita income of up to one-half of the minimum wage or a total family income of three minimum wages. The system collects social identification information for each family member and information regarding their income and living conditions. (Brasil, 2017) The baseline of the 100 M Cohort consists of all individuals applying for the first time to CadÚnico.

The 100 M Cohort baseline was linked to individual-level mortality records from the Mortality Information System using a non-deterministic matching procedure based on the individual’s name, mother’s name, sex, year of birth, and municipality of residence using the Cidacs-RL software (Barbosa et al., 2020) and done in the Center of Data and Knowledge Integration for Health (CIDACS) safety environment. (Barreto et al., 2019).

Ethical approval for this study was obtained from the Research Ethics Committees of the Institute of Collective Health at the Federal University of Bahia and the Gonçalo Moniz Institute of the Oswaldo Cruz Foundation.

### 2.2 Outcome data

We included all non-accidental deaths among participants of the 100 M Cohort. Mortality records were coded according to the International Classification of Diseases, 10th Revision (ICD-10). We included all codes from A00 to R99, excluding external/accidental causes of mortality. Deaths with invalid or missing information on the municipality of residence and date of death were excluded (see File S1). Finally, individual death records were daily aggregated at the municipality of residence.

### 2.3 Exposure data

Daily minimum and maximum ambient temperature were obtained from the Brazilian Daily Weather Gridded Data (BR-DWGD), available at a 0.1° × 0.1° spatial resolution. (Xavier et al., 2022) The BR-DWGD temperature series were estimated by interpolation using data from 1,252 meteorological stations of the Brazilian National Institute of Meteorology. The interpolation was performed using the Inverse Distance Weighting method, adjusting the query point’s elevation and a temperature lapse rate correction to reduce thermal discrepancies caused by topographical variations. The interpolation demonstrated good performance in cross-validation, with an RMSE of 1.557 °C for maximum temperature and 1.616 °C for minimum temperature. (Xavier et al., 2022).

We estimate the daily mean temperature as the average of the maximum and minimum temperatures recorded on a given day. Finally, we generated a municipality-level daily time series of mean temperature by calculating the area-weighted average of grid values, where weights correspond to the fraction of the municipality’s area covered by each grid cell.

### 2.4 Potential effect modifiers

Potential effect modifiers were selected based on the literature on climate vulnerability and resilience. (Abbass et al., 2022; Son et al., 2019; Benmarhnia et al., 2015; Gronlund, 2015) The identification of these factors followed a conceptual framework encompassing environmental, household, and individual characteristics that may influence the relationship between temperature and mortality (File S2). These factors include environmental characteristics that may affect the intensity of extreme temperature events, as well as social determinants and characteristics of the household that may contribute to exposure disparities. Individual-level factors (e.g., age) may also be related to physiological susceptibility and thermoregulatory capacity.

The final set of variables included age (<60 years, ≥60 years), sex (men, women), race/color (Black, White), family per capita income, geographic location (region and urban–rural residence), and access to essential utilities.

Race was used as a proxy for exposure to systemic racism. (Lett et al., 2022) In Brazil, race/color is self-reported and categorized as Indigenous, Black, Parda (Brown), Asian, and White. (Petruccelli and Saboia, 2013) The Parda category, considered part of the Afro-descendant population, includes people of mixed racial backgrounds and is also affected by racism. Parda and Black individuals were grouped together and referred to collectively as Black. The Asian and Indigenous categories were excluded from the effect modification analysis due to reduced sample sizes and regional inconsistencies in self-identification. (Petruccelli and Saboia, 2013).

The family per capita income was analyzed in tertiles and calculated by dividing the total earnings from work, donations, pensions, and other benefits of all family members by the number of people living in the household. (Brasil, 2017).

Access to key utilities included water supply (public network, well or natural source, cistern, or others), waste collection (public collection system, burned or buried, outdoor disposal, or other), and electricity access (home meter, community meter, irregular electricity, gas lighting, candlelight, or other). These essential utilities were categorized as having all services provided through public or private infrastructure or lacking one or more essential services.

After the potential effect modifiers selection, we generated a daily municipal-level aggregated times series of non-accidental deaths for each subgroup (e.g., a daily municipal-level times series of deaths for females and another for males). We build these subgroup time-series considering complete case data, i.e., for those individuals in the cohort we have the information available about that subgroup.

## 3 Statistical analyses

We investigated the effects of ambient temperature on mortality using a space–time-stratified case-crossover design. The space–time-stratified case-crossover design extends the traditional case-crossover approach by allowing multilocation data to be analyzed in one stage, rather than through a two-stage procedure, while incorporating temporal and spatial matching to control for time-invariant confounders and account for regional variations. (Wu et al., 2021).

In all analyses, the outcome was defined as the daily count of non-accidental deaths aggregated at the municipality level. We used a space–time-stratified referent selection, with strata defined by municipality, year, month, and day of the week. Each case day was therefore matched to control days occurring in the same municipality, year, and month, and on the same day of the week. This approach controls long-term trends, seasonality, day-of-week effects, and spatial heterogeneity. (Wu et al., 2021; Tobias et al., 2024).

The effects were estimated using conditional quasi-Poisson regression models combined with distributed lag non-linear models (DLNMs). (Armstrong et al., 2014; Gasparrini, 2011) The quasi-Poisson distribution was chosen to deal with overdispersion and the DLNM to account for non-linear and lagged effects of ambient temperature and mortality. The specification of DLNMs parameters requires determining the number of lags to account for the delayed effects of temperature and selecting the appropriate functional forms for both the exposure–response and lag–response relationships. We used a 21-day lag period to capture both heat effects (typically within a few days) and cold effects (which can extend up to several weeks). (Gasparrini et al., 2015; Guo et al., 2014).

Given Brazil’s climatic heterogeneity and demographic diversity, model selection was conducted separately for each of Brazil’s five macro-regions (North, Northeast, Central-West, Southeast, and South). Detailed model specification criteria are provided in the supplementary material (File S3). Briefly, we tested multiple specifications for the exposure–response dimension (i.e., temperature), varying the number and placement of knots. Final model selection was guided by prior studies conducted in Brazil (Aschidamini and Ponce de Leon, 2025; Silveira et al., 2019), the shape of exposure–response relationships, and model fit assessed through the quasi-likelihood Akaike Information Criterion (qAIC) (File S3).

Potential effect measure modification was investigated through stratified analyses, i.e., running separated models for each time series of interest. Relative risks (RRs) and confidence intervals (CIs) were estimated for cold-related mortality at the first percentile and heat-related mortality at the 99th percentile of regional temperature distributions compared to the region-specific minimum mortality temperature (MMT). For the North region, since the estimated regional MMT exceeded the 99th percentile, the MMT was defined using the national MMT percentile as the basis for calculation (File S3). (Guo et al., 2014).

Sensitivity analyses were conducted to assess the robustness of the results, including analysis with a shorter lag period (6 days) in the main models for each region and Brazil; and different specifications of the number and position of knots in the temperature cross-basis (File S3).

All statistical analyses were performed in R software. The R codes and data files not subject to data protection Brazilian regulations are available at: https://github.com/reprotc/Temperature_100MCohort.

## 4 Results

The characteristics of our study population are shown in Table 1, including 3,554,422 non-accidental deaths, after excluding 3,395 records due to missing data on the municipality of residence or date of death (File S1). Men comprised 52 % of deaths, while 56 % occurred among individuals aged 60 years or older and 63 % among Black individuals. Most of the study population lived in urban areas (78 %). Among the 44 % of the study population with complete information regarding access to essential utilities, 59 % lacked at least one basic service. The missing data among the potential effect modifiers were low, except for essential utilities (Table 1).

National and regional descriptive statistics of daily mean temperatures during the study period (2000–2018) are presented in Table S4, along with the Köppen-Geiger climate classification map (Fig. S4). Nationally, temperatures across 5,570 Brazilian municipalities ranged from −3°C to 35 °C with a mean of 24 °C. At the regional level, temperatures ranged from 14 °C to 35 °C in the North (tropical rainforest climate), 13 °C to 35 °C in the Northeast (semi-arid and tropical climates), 6 °C to 35 °C in the Central-West (tropical savanna climate), 4 °C to 34 °C in the Southeast (mixed subtropical climates), and −3°C to 34 °C in the South (humid subtropical climate). Corresponding interquartile ranges were 5 °C (Brazil), 2 °C (North), 3 °C (Northeast), 3 °C (Central-West), 5 °C (Southeast), and 7 °C (South).

**Fig. 1:**
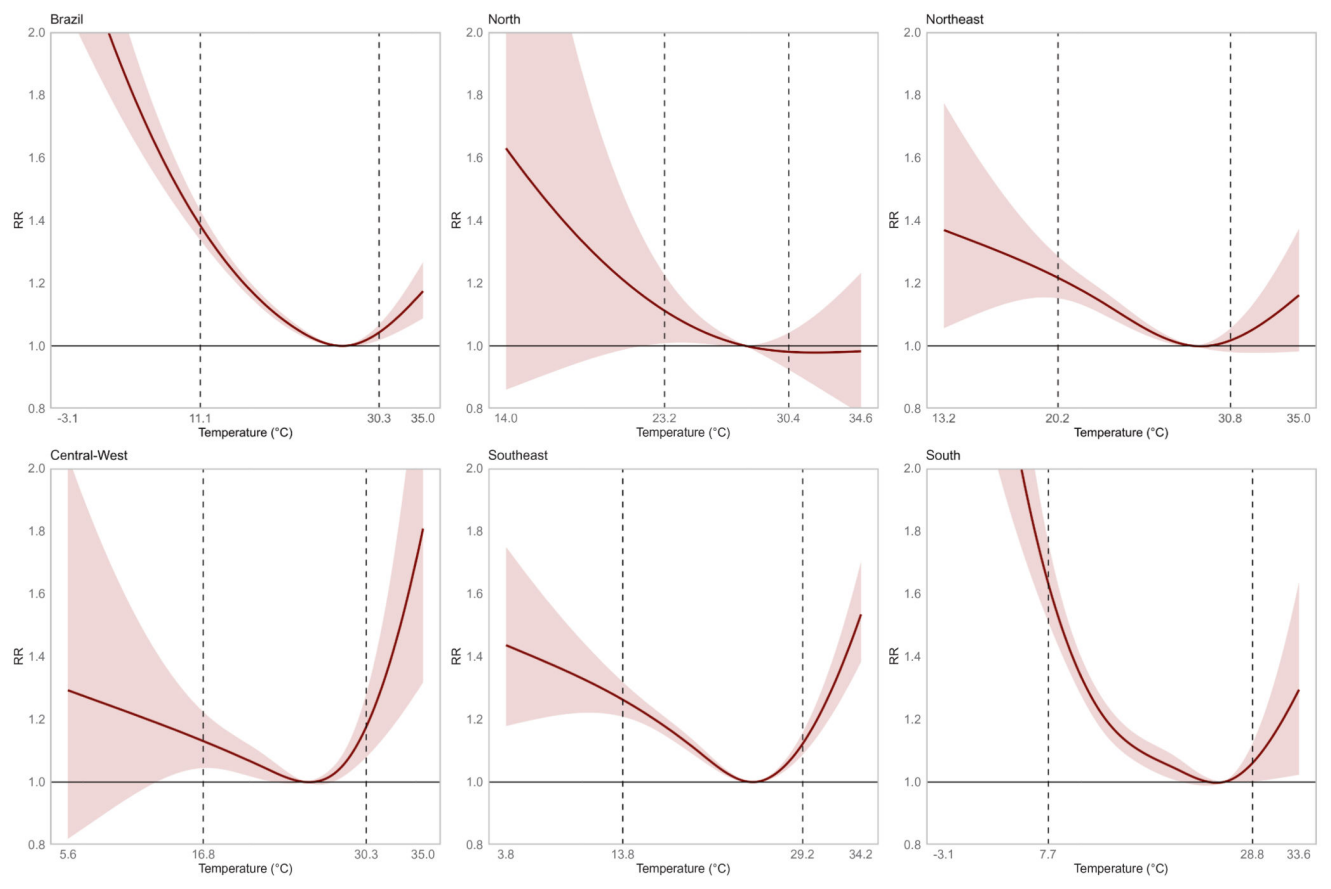
Association between ambient temperature and non-accidental mortality in the 100 Million Brazilian Cohort, Brazil and macro-regions (2000–2018). The shaded areas represent the 95% confidence intervals, and the dotted lines indicate the 1st (cold) and 99th (heat) percentiles of the regional mean temperature distribution. The range of daily mean temperature is also shown on the x-axis.

**Fig. 2:**
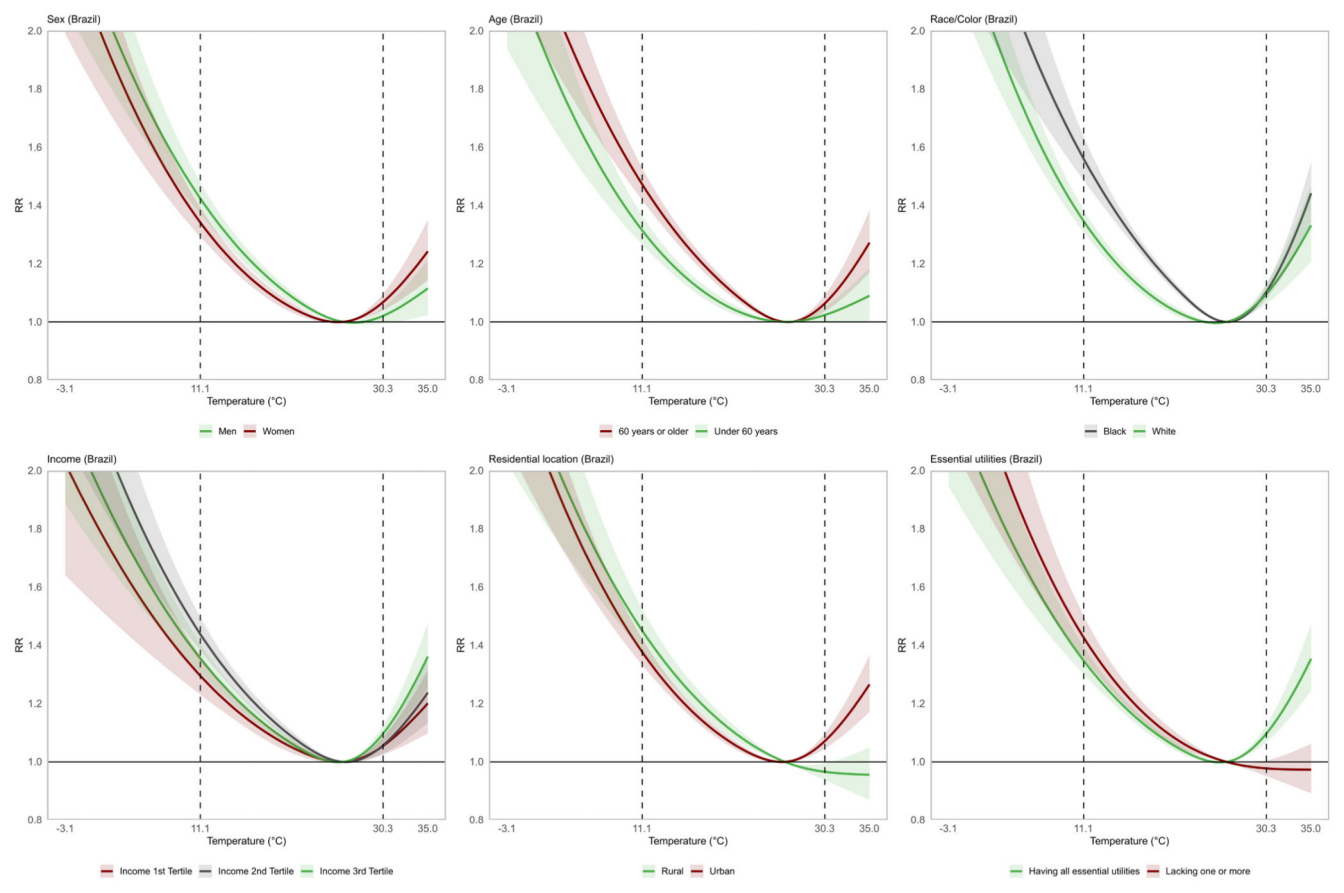
Association between ambient temperature and non-accidental mortality among population subgroups from the 100 Million Brazilian Cohort (2000–2018). The shaded areas represent the 95% confidence intervals, and the dotted lines indicate the 1st (cold) and 99th (heat) percentiles of the regional mean temperature distribution. The range of daily mean temperature is also shown on the x-axis.

The estimated temperature-mortality relationship for Brazil and its five macro-regions is shown in Fig. 1. The relationship follows an inverted J-shaped pattern at the national level, with increased mortality risk at lower and higher temperatures and a more pronounced risk at lower temperatures. Regional heterogeneity was observed, with the South exhibiting the highest cold-related mortality risk. Similarly, cold-related mortality was more pronounced in the North and Northeast compared to heat-related mortality risk. In contrast, the temperature-mortality relationship in the Southeast followed a more distinct U-shaped pattern, with increased mortality risk at lower and higher temperatures. The exposure–response curves for most population subgroups resembled the inverted J-shaped pattern observed for the overall population (Fig. 2).

Table 2 presents the cumulative relative risks (RRs) of non-accidental mortality associated with cold (1st percentile x MMT) and heat (99th percentile x MMT) for Brazil overall and stratified by macro-regions and population subgroups among the 100 million Brazilian cohort from 2000 to 2018.

At the national level, cold temperatures were associated with a 38 % increase in risk of non-accidental mortality (RR = 1.38, 95 % CI: 1.34–1.43). Regional heterogeneity was observed, with the South exhibiting the highest cold-related mortality risk (RR = 1.63, 95 % CI: 1.51–1.76), followed by the Southeast (RR = 1.26, 95 % CI: 1.21–1.32), Northeast (RR = 1.22, 95 % CI: 1.15–1.29), Central-West (RR = 1.13, 95 % CI: 1.04–1.22), and the North (RR = 1.11, 95 % CI: 1.01–1.23).

Cold-related mortality also varied across population groups. At the national level, mortality risk associated with cold temperatures increased by 47 % (RR = 1.47, 95 % CI: 1.42–1.53) among older adults (≥ 60 years) and by 31 % (RR = 1.31, 95 % CI: 1.27–1.36) among those under 60 years. Racial differences were also observed, with an increase in cold-related mortality risk by 56 % (RR = 1.56, 95 % CI: 1.49–1.64) among Black individuals, which was higher than the risk observed among White individuals (RR = 1.35, 95 % CI: 1.31–1.38).

In contrast, we found an unexpected pattern for cold-related mortality across income strata, with the intermediate income group (second tertile) showing higher mortality risk (RR = 1.44, 95 % CI: 1.38–1.50), relative to the lowest income group (RR = 1.30, 95 % CI: 1.23–1.36).

We found no evidence of effect modification of cold-related mortality by sex, residential location (urban and rural), and essential utilities, with all subgroups showing comparable increased risk of mortality associated with cold temperatures.

At the national level, heat was associated with a 4 % increase in mortality risk (RR = 1.04, 95 % CI: 1.02–1.07). Regional patterns differed markedly from cold effects, with only the Southeast (RR = 1.22, 95 % CI: 1.09–1.58) and Central-West regions (RR = 1.18, 95 % CI: 1.08–1.28) showing statistically significant increases in heat-related mortality risk.

Similarly, heat vulnerability patterns across population subgroups differed from those observed for cold temperatures. While we found no consistent evidence of effect modification by age and sex, a significant increased risk was observed among women (RR = 1.07, 95 % CI: 1.04–1.10) and older adults (RR = 1.06, 95 % CI: 1.04–1.09). In contrast, both White and Black individuals showed similar heat-related mortality risk (RR = 1.10, 95 % CI: 1.06–1.13 and RR = 1.10, 95 % CI: –1.13, respectively).

Regarding residential location, increased heat-related mortality risk was found among urban residents (RR = 1.07, 95 % CI: 1.05–1.10), in contrast with a slight decrease in risk among rural residents (RR = 0.97, 95 % CI: 0.94–0.99).

An unexpected pattern was observed for access to essential utilities (electricity, water supply, and waste collection), with statistically significant increase in heat-related mortality risk found only among individuals with access to all basic services (RR = 1.10, 95 % CI: 1.07–1.13).

Sensitivity analyses suggest the stability of effect estimates across different model specifications (File S4). Overall, estimates were similar when varying the number and position of spline knots. However, for analyses with shorter lag periods (0–6 days), we found an expected reduction in effect estimates for cold-related mortality across all regions and higher heat effects, particularly for Brazil overall, Central-West, Southeast, and South regions.

## 5 Discussion

Using nationwide data from the 100 Million Brazilian Cohort, our findings show increased cold- and heat-related mortality risk, with a more pronounced risk at lower temperatures. Regional heterogeneity was observed, with the South exhibiting the highest mortality risk associated with cold temperatures, while significant increases in heat-related mortality risk were found only in the Southeast and Central-West regions. Our results indicate increased vulnerability to cold-related mortality among older adults and Black individuals. Our results also suggest higher vulnerability to heat-related among urban residents. However, we found no consistent evidence of effect modification of cold- and heat-related mortality by sex, income and essential utilities.

Our findings of higher cold-related mortality among Black populations are consistent with a prior study in US (Liu et al., 2025) but differ from other studies that documented higher heat-related mortality risks among historically marginalized racial groups. (Son et al., 2019; Keivabu et al., 2024) Racial differences in temperature-related mortality likely reflect socioeconomic inequalities, exposure disparities, higher prevalence of chronic diseases, and differences in adaptive capacities. In turn, these proximal factors are shaped by both historical and ongoing processes, including systemic racism and the persistent effects of environmental degradation affecting marginalized communities. (Gronlund, 2015).

Similar to our findings, a previous study conducted in Brazilian metropolitan areas showed greater cold-related vulnerability among older adults and found no evidence of age differences in heat-related mortality risk (Aschidamini and Ponce de Leon, 2025). However, evidence of elevated mortality risks for cold and heat exposure among older adults has been reported globally. (Benmarhnia et al., 2015) The higher vulnerability observed among older adults is often attributed to physiological factors such as reduced thermoregulatory capacity, higher prevalence of chronic diseases, medications that interfere with heat dissipation, and mobility impairments. (Bell et al., 2024).

While we found no evidence of effect modification by sex, studies conducted in multiple continents, (Son et al., 2019) including low- and middle-income countries, (Green et al., 2019) indicate increased vulnerability to heat-related mortality among women. Although sex differences are often explained by physiological or socioeconomic factors, variations in age distribution across sex groups in prior studies may also contribute to these observed disparities. (Benmarhnia et al., 2015) Supporting our findings, a large cohort study of Chinese older adults also found no significant sex differences in temperature-related mortality (Xi et al., 2024).

Few studies in low- and middle-income countries have assessed the role of individual-level socioeconomic factors as modifiers of temperature effects on mortality. Among these studies, educational attainment was the predominant socioeconomic measure, showing increased vulnerability to heat among individuals with lower education levels. (Green et al., 2019; Benmarhnia et al., 2015) In contrast, studies using area-level income measures have reported mixed findings, with some indicating greater heat-related vulnerability in areas with higher income inequality, (Sera et al., 2019) while others have found unexpectedly lower risks in the lowest-income areas. (Bakhtsiyarava et al., 2023) Although we found no consistent evidence of effect modification of temperature-related mortality by income per se, the 100 M Cohort exclusively comprises low-income individuals, which may reduce the meaningful contrasts between income groups. We hypothesized that this lack of contrast could also have affected the power to detect a potential effect modification by race/color and sex. Additionally, the income composition of our study population may partly explain our higher estimates for non-accidental mortality compared to other studies conducted in Brazil and Latin America.(Bakhtsiyarava et al., 2023; Kephart et al., 2022).

Overall, evidence of urban–rural disparities in temperature-related mortality remains inconsistent. (Son et al., 2019; Sera et al., 2019) Our results align with some studies that report higher heat-related mortality in urban settings in countries such as Switzerland and England, (de Schrijver et al., 2023; Gasparrini et al., 2022) while contrasting with others that suggest greater vulnerability among rural populations in countries such as China and South Korea. (Hu et al., 2019; Lee et al., 2022) Other findings indicate that both highly urbanized and sparsely populated rural areas experience greater vulnerability to heat-related mortality. (Hu et al., 2019) A potential limitation of our findings relates to using municipal-level exposure data in our analyses, which could have masked disparities in exposure between urban and rural residents. Additionally, while urban residents face different risk factors, such as the urban heat island effect, rural populations may experience greater vulnerability due to extended outdoor activities and limited infrastructure. The interplay of these distinct factors may add complexity to evaluating disparities in heat-related mortality across urban and rural settings, together with the heterogeneous definition of what constitutes an urban and rural location. (Tonne et al., 2021).

Several factors may have contributed to the regional disparities in temperature-related mortality risk observed in our study. Socioeconomic conditions, population characteristics, and data quality are key considerations. Regional differences could reflect unique local vulnerabilities, including inadequate housing, limited healthcare access, and pronounced socioeconomic inequalities. Differences in mortality data quality between regions may have also contributed to variations in estimated risks. Although mortality data in Brazil are generally of good quality, regional differences exist. The North and Northeast show lower data completeness and coverage below national standards. (Rebouças et al., 2025) Similarly, as observed in many other countries, Brazil’s spatial coverage of weather stations is irregular, with pronounced regional differences and sparse coverage in the northern region. These limitations may have led to misclassification of the exposure–response relationship, especially in the North. Additionally, regions with lower temperature variability may present reduced contrast between case and control periods, potentially leading to underestimation of effect estimates. Another point is that smaller sample sizes for regions like the North. On the other hand, some counterintuitive behaviors could also reflect long-term acclimatization and adaptation strategies that mitigate temperature-related effects. Future research should further explore the role of population adaptive capacity in assessing vulnerability to ambient temperatures.

Our results should be interpreted in light of some limitations. The use of municipality-level exposure data may have masked differences in risk among population subgroups, particularly those arising from disparities in individual exposure, which are not fully captured when using temperature data with coarser spatial resolution. Other determinants of individual exposure, such as personal mobility and air conditioning and heating device possession, were also not considered in our exposure assessment. Additionally, while we performed region-specific models to account for Brazil’s climatic heterogeneity, residual within-region variability between cities may persist due to local factors such as urban heat island effects and varying socioeconomic conditions. The proportion of missing data in variables traditionally challenging to collect in epidemiological studies, particularly socioeconomic and infrastructure factors, may also account for some inconsistent findings, such as for essential utilities. The potential impact of sample size for some analysis also reflects our study design, which does not leverage the full statistical power of the 100 M Cohort but only selected cases and matched control periods. Nevertheless, with ongoing geocoding and high-resolution exposure assessments, the 100 M Cohort likely represents one of the largest cohorts for environmental health and climate change investigations.

Our findings add to the growing evidence base from low- and middle-income countries, particularly in Latin America. We identified significant regional differences, highlighting populations particularly vulnerable to temperature-related mortality. This evidence could help design targeted interventions to protect vulnerable populations from extreme temperature events and effective adaptation strategies.

## Supplementary Material

Supplementary Materials

## Figures and Tables

**Table 1:** Summary statistics of non-accidental mortality cases within the 100 Million Brazilian Cohort. 2000–2018.

	Brazil N (%)	North N (%)	Northeast N (%)	Central-West N (%)	Southeast N (%)	South N (%)
**Non-accidental mortality**	3,554,422	238,085	1,325,159	237,339	1,247,696	506,138
**Sex**						
Women	1,703,392 (47.9)	112,270 (47.2)	636,304 (48)	112,457 (47.4)	604,695 (48.5)	237,664 (47)
Men	1,851,030 (52.1)	125,815 (52.8)	688,855 (52)	124,882 (52.6)	643,001 (51.5)	268,474 (53)
*Missing*	0	0	0	0	0	0
**Age**						
Under 60 years	1,572,503 (44.3)	113,045 (47.6)	587,975 (44.5)	98,010 (41.4)	566,481 (45.5)	205,274 (40.6)
60 years or older	1,976,255 (55.7)	124,397 (52.4)	732,868 (55.5)	138,811 (58.6)	679,210 (54.5)	300,094 (59.4)
*Missing*	0.2	0.3	0.3	0.2	0.2	0.2
**Race**						
White	1,173,758 (36.2)	26,950 (12.4)	226,068 (18.8)	68,589 (31.2)	502,732 (44)	349,415 (76.3)
Black	2,038,735 (62.9)	186,417 (85.5)	963,508 (80.3)	147,046 (66.8)	634,428 (55.5)	105,550 (23.1)
Indigenous	14,840 (0.5)	3,785 (1.7)	4,563 (0.4)	2,971 (1.3)	1,908 (0.2)	1,613 (0.4)
Asian	13,472 (0.4)	924 (0.4)	5,516 (0.5)	1,517 (0.7)	4,397 (0.4)	1,118 (0.2)
*Missing*	8.8	8.4	9.5	7.3	8.4	9.6
**Income** *						
1st tertile (lowest)	785,603 (33.3)	53,565 (33.3)	283,708 (33.3)	55,375 (33.3)	280,203 (33.3)	112,751 (33.3)
2nd tertile	785,597 (33.3)	53,564 (33.3)	283,707 (33.3)	55,374 (33.3)	280,202 (33.3)	112,750 (33.3)
3rd tertile (highest)	785,597 (33.3)	53,564 (33.3)	283,707 (33.3)	55,374 (33.3)	280,202 (33.3)	112,750 (33.3)
*Missing*	5.5	5.8	3.5	6	7.5	5
**Residential location**						
Urban	2,686,997 (77.9)	168,026 (73.4)	858,567 (67.1)	200,866 (87)	1,051,919 (86.6)	407,616 (82.7)
Rural	760,416 (22.1)	60,837 (26.6)	420,609 (32.9)	30,086 (13)	163,336 (13.4)	85,546 (17.3)
*Missing*	3	3.9	3.5	2.7	2.6	2.6
**Essential utilities**						
Having all essential utilities	633,266 (40.9)	29,568 (23.3)	131,459 (21.2)	57,684 (60.2)	312,724 (60.4)	101,830 (53.9)
Lacking one or more	916,003 (59.1)	97,194 (76.7)	488,660 (78.8)	38,083 (39.8)	205,075 (39.6)	86,989 (46.1)
*Missing*	56.4	46.8	53.2	59.6	58.5	62.7

* Data on income were restricted to the 2011–2018 period, with missing data percentages calculated for this period. Missing data for all other variables are presented as percentages of total non-accidental deaths over the entire study period (2000–2018).

**Table 2:** Mortality risks associated with cold (1st percentile) and heat (99th percentile) across macro-regions and population subgroups from the 100 Million Brazilian cohort, Brazil (2000–2018).

	Group	RR_cold_	95 %CI_cold_	RR_Heat_	95 %CI_Heat_
	Brazil	1.38	(1.34–1.43)	1.04	(1.02–1.07)
Macro-region	North	1.11	(1.01–1.23)	0.98	(0.92–1.04)
	Northeast	1.22	(1.15–1.29)	1.02	(0.98–1.06)
	Central-West	1.13	(1.04–1.22)	1.18	(1.08–1.28)
	Southeast	1.26	(1.21–1.32)	1.22	(1.09–1.58)
	South	1.63	(1.51–1.76)	1.06	(1.00–1.12)
Sex	Men	1.42	(1.37–1.48)	1.02	(0.99–1.05)
	Women	1.34	(1.29–1.39)	1.07	(1.04–1.10)
Age	Under 60 years	1.31	(1.27–1.36)	1.02	(1.00–1.05)
	60 years or older	1.47	(1.42–1.53)	1.06	(1.04–1.09)
Race/color	White	1.35	(1.31–1.38)	1.10	(1.06–1.13)
	Black	1.56	(1.49–1.64)	1.10	(1.08–1.13)
Income	Income 1st tertile	1.30	(1.23–1.36)	1.05	(1.02–1.08)
	Income 2nd tertile	1.44	(1.38–1.50)	1.06	(1.03–1.09)
	Income 3rd tertile	1.36	(1.31–1.41)	1.10	(1.07–1.13)
Residential location	Urban Rural	1.38 1.45	(1.33–1.42) (1.38–1.53)	1.07 0.97	(1.05–1.10) (0.94–0.99)
					
Essential utilities	Having all essential utilities	1.35	(1.30–1.40)	1.10	(1.07–1.13)
	Lacking one or more	1.43	(1.36–1.50)	0.98	(0.95 – 1.00)

## Data Availability

The R code and data files not subject to Brazilian data protection laws are available at: https://github.com/reprotc/Temperature_100MCohort
