# Supplementary material for: Ambient temperature and non-accidental mortality: A nationwide space–time-stratified case-crossover study within the 100 million Brazilian Cohort
Source: Environ Int. Author manuscript; Available in PMC 2025 Dec 22. (PMC7618503; doi:10.1016/j.envint.2025.109892)
Supplement: Supplementary Materials [file EMS211427-suppement-Supplementary_Materials.pdf]

Appendix A. Supplementary data

Supplementary data to this article can be found online at https://doi.org/10.1016/j.envint.2025.109892.
